# The “Lasso Sign”: An Early Sonographic Sign of Posterior Meningocele

**DOI:** 10.5402/2011/175916

**Published:** 2010-10-26

**Authors:** Alina Weissmann-Brenner, Zeev Feldman, Yaron Zalel

**Affiliations:** ^1^Department of Obstetrics and Gynecology, The Chaim Sheba Medical Center, Tel HaShomer 52621, Israel; ^2^Pediatric Neurosurgery Unit, The Edmond and Lily Safra Children's Hospital, The Chaim Sheba Medical Center, Tel HaShomer 52621, Israel

## Abstract

Posterior meningocele is an uncommon form of spina bifida. We present a case of unique posterior meningocele diagnosed at the early second trimester anatomical scan using 2D and 3D ultrasound. The sonographic appearance resembled “lasso”. The prenatal follow-up was uneventful, with no demonstration of tethered cord. Clinical, neurological and radiological examinations following delivery and at the age of four months were unremarkable.

The differential diagnosis for a dorsal midline mass presenting in fetuses and newborns includes a wide range of pathological conditions, consisting of spinal dysraphisms, teratoma, hamartoma, disturbances in regression of fetal tail, and pseudotail formation [1].

Posterior meningocele is the least common form of spina bifida. It appears as a sac or cyst which contains cerebrospinal fluid. The majority of cases have no underlying bony defect or communication to the meninges. The spinal cord and nerves are not involved and their function is normal. Its incidence is 1 : 1000 live births [2, 3].

Apart from spina bifida, causes of meningocele include teratoma and other tumors of the sacrococcyx and of the presacral space and Currarino syndrome. Usually a meningocele has no negative long-term effects, although there are reports of tethered cord. 

We present a case of unique posterior meningocele diagnosed at the early second trimester anatomical scan using 2D and 3D ultrasound.

A healthy 29-year-old woman presented to our ultrasound unit at her first pregnancy for a routine anatomical scan at 15-week gestation. 

The anatomical scan revealed a small cystic lesion with very tiny stalk close to the caudal aspect of the spine, “floating" in the amniotic fluid, with no flow when Doppler was applied. This lesion was suspected to be meningocele, and based on its appearance we have termed it as the “Lasso sign". No opened spina bifida was noted. 3D ultrasound using sectional planes and surface rendering was performed, and it demonstrated the same lesion and confirmed it to be meningocele alone. The anatomical scan of the brain including the posterior fossa was normal. 

During the present pregnancy she performed nuchal translucency of 0.8 mm.

The triple test screen was normal and consisted of alpha-fetoprotein level of 0.95 MOM. The patient did not perform MRI due to claustrophobia.

One of the complications of meningocele is tethered cord, caused by pathological fixation of the spinal cord, resulting in traction on the neural tissue which, in turn, leads to ischemia and progressive neurological deterioration. The prenatal diagnosis of tethered cord is based on ultrasound evaluations of the spinal cord and the conus medullaris at the level of L2-L3 at the second and third trimester [4, 5]. Repeated sonograms in our case demonstrated the same lesion at L3-L4 (see Figure 1 and supplementary movie Lasso Movie 1 in Supplementary Material available online at doi:10.5402/2011/175916) without sonographic signs of tethered cord. 

At 40 + 2 weeks of gestation the patient underwent a cesarean section because of arrest of dilatation. A 3384gr female infant was delivered with Apgar scores of 9 and 10 at 1 minute and 5 minutes, respectively.

The neonate's physical exam revealed an atretic string of about 2 cm on the lower aspect of her back in the midline, confirmed by a neurosurgeon to be meningocele. Additional evaluation included ultrasound of the brain which was normal, ultrasound of the sacrum which ruled out tethered cord, and X-ray of the spine which demonstrated normal structure of the vertebrae. Movements of four limbs and muscle tone were normal. There was no leakage of urine, and the tonus of the anal sphincter was good. The string dried out and fell 12 hours after birth. Pathological evaluation was not performed; therefore, the diagnosis was based on the prenatal ultrasound performed by gynecologists and pediatric neurosurgeons and by the clinical examination after birth by the pediatric neurosurgeon. Photos of the back of the infant taken at the age of 2 days were unremarkable, apart from a tiny scar at the middle of her lower back that disappeared after a few days. Followup examination including neurological examination at the age of two months and four months was unremarkable. MRI performed at the age of four months was normal.

In summary, detection of the “Lasso” sign may indicate the diagnosis of posterior meningocele. Thorough evaluation of the fetal brain and spinal cord to exclude Chiari II malformation and spina bifida is mandatory. The use of colour Doppler to rule out the possibility of umbilical cord as part of the examination is necessary. Level of alpha-fetoprotein should be examined. Prenatal followup should include repeated sonographic evaluation of the brain and spinal cord with emphasis on the conus medullaris to rule out tethered cord. 3D ultrasound may aid both in the diagnosis and followup. MRI may also be offered in order to aid in the diagnosis. In general, posterior meningocele has no long-term effect. “Lasso" appearing meningocele should be included in the differential diagnosis of lower spine posterior cystic lesions.

## Figures and Tables

**Figure 1:**
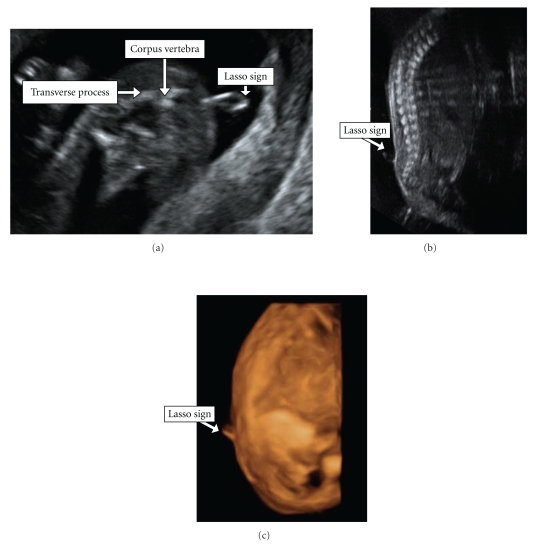
(a) Transverse 2D views of the lower spine revealing posterior meningocele at 15 weeks. (b) Sagittal 2D view of the posterior meningocele at 22 + 3 weeks. (c) Sagittal 3D view of the posterior meningocele at 22 + 3 weeks.

## References

[1] Amirjamshidi A, Abbassioun K, Bidabadi MS (2006). Skin-covered midline spinal anomalies: a report of four rare cases with a discussion on their genesis and milestones in surgical management. *Child’s Nervous System*.

[2] El Shabrawi-Caelen L, White WL, Soyer HP, Kim B-S, Frieden IJ, McCalmont TH (2001). Rudimentary meningocele: remnant of a neural tube defect?. *Archives of Dermatology*.

[3] Klble N, Huisman TAGM, Stallmach T, Meuli M, Zen Ruffinen Imahorn F, Zimmermann R (2001). Prenatal diagnosis of a fetus with lumbar myelocystocele. *Ultrasound in Obstetrics and Gynecology*.

[4] Robbin ML, Filly RA, Goldstein RB (1994). The normal location of the fetal conus medullaris. *Journal of Ultrasound in Medicine*.

[5] Zalel Y, Lehavi O, Aizenstein O, Achiron R (2006). Development of the fetal spinal cord: time of ascendance of the normal conus medullaris as detected by sonography. *Journal of Ultrasound in Medicine*.

